# Enhancing the quality of rice-based gluten-free bread using sourdoughs fermented with *Lactobacillus fermentum* and *Lactobacillus plantarum*

**DOI:** 10.1038/s41598-025-11872-4

**Published:** 2025-07-22

**Authors:** Sedigheh Seyedahmadi, Mehdi Gharekhani, Sepideh Tariverdi, Hamid Bakhshabadi

**Affiliations:** 1 Department of Biological Sciences, Tabriz Higher Education Institute of Rab- Rashid, Tabriz, Iran; 2 Department of Food Science and Technology, Ta.C., Islamic Azad University, Tabriz, Iran; 3 Department of Agriculture, Minab Higher Education Center, University of Hormozgan, Bandar Abbas, Iran

**Keywords:** Gluten-free bread, Rice, Sourdough, Lactobacillus, Biochemistry, Chemistry

## Abstract

This study focused on enhancing the quality of gluten-free rice flour bread by incorporating sourdough fermented with two specific bacterial strains, *Lactobacillus fermentum* and *Lactobacillus plantarum*. The research examined the impact of the bacterial type and dough yield (200% and 300%) on sourdough characteristics such as pH, lactic acid concentration, and lactic acid bacteria count. Sourdough prepared at three different concentrations (10%, 20%, and 30%) was then used in baking rice-based gluten-free bread. The results indicated that the highest pH in sourdough (4.53 and 4.60) was associated with samples using a *L. fermentum* starter at 200% and 300% yield. Meanwhile, the sample made with *L. plantarum* at 200% yield had the highest lactic acid content (620.33 mg/100 g dry basis). Additionally, an increase in sourdough content in the bread formula resulted in lower pH levels, mold and yeast count, and a* and b* color indices, but higher moisture content and L* color index. A rise in dough yield led to increased pH, baking loss, and mold and yeast count. The highest mold and yeast counts (7.95 log cfu/g) were found in control bread after four days of storage, while the lowest counts (0.72 log cfu/g) were observed in bread made with 30% sourdough containing either bacterial strain at a 200% dough yield on the first day. The sensory acceptance peaked and then decreased with sourdough levels up to 20%. The optimal bread, based on sensory evaluation, was made with 20% sourdough using either *L. fermentum* or *L. plantarum* at a 200% dough yield, highlighting the benefits of specific sourdough concentrations and bacterial strains in improving gluten-free bread.

## Introduction

One of the most consumed grain-based products is bread, the quality of dough from wheat flour is attributed to the unique properties of gluten proteins. Gluten proteins contribute to the stickiness and development of wheat dough, with the glutenin part of gluten playing a crucial role in the dough’s strength and elasticity. Gluten formation occurs when flour is mixed with water. Despite its insolubility and hydrophobicity, gluten can absorb almost twice its weight in water^1^. On the other hand, in the past two decades, the demand for gluten-free products has increased due to the rising trend in the diagnosis of celiac disease worldwide^2^. Celiac disease is a gastrointestinal disorder that can overlap with several other gastrointestinal conditions, including functional bowel disorders, lactose or fructose intolerance, pancreatic insufficiency, and others. The consumption of gluten by individuals with celiac disease causes inflammation and swelling of the small intestine, leading to malabsorption of nutrients and vitamins, as well as potential gastrointestinal bleeding, fever, sweating, and intestinal blockage^3^. To effectively manage celiac disease, the only proven remedy is a lifelong commitment to a diet devoid of gluten^4^. For the production of gluten-free bread, it’s essential to use flours that are alternatives to wheat flour. Rice stands out as a crucial grain and staple food item globally, with half of the global population relying on it as their primary food source. The world hosts thousands of rice varieties, broadly classified into two major subspecies: the “Japonica” variety, scientifically referred to as *Oryza sativa* japonica, and the “Indica” variety, known scientifically as *Oryza sativa* indica^5^. Healthy food consumption has emerged as a significant trend in contemporary society, reflecting people’s efforts for health and longevity. It is very important to maintain a healthy lifestyle. On the other hand, studies have shown that products labeled as"additive-free” are expected to be healthier and more palatable than products containing additive ingredients^6,7^. Among the challenges of the modern food industry, the production of sourdough bread to enhance sensory properties, nutrition, and shelf life is a response to the increasing demand from consumers for additive-free, safe, and nutritious food. Additionally, the wastage of bread due to staleness and microbial spoilage (mainly mold and stringiness) leads to significant economic losses^8,9^. Innovations in bread production over the past decade have included the use of enzymes, natural preservatives primarily produced by lactobacilli, and sourdough technology. Undoubtedly, the use of various sourdoughs, which have been utilized as natural leavening agents since ancient times, has yielded highly successful outcomes. Sourdough offers numerous benefits, such as enhancing the nutritional value and health aspects of bread^10^. Essentially, sourdough is a mixture of water and grain flour that constitutes a highly complex and sterile fermentative biological system, which can be used as a natural additive in bread making. The most crucial factors influencing sourdough fermentation include the type of starter, fermentation conditions (time and temperature, number of cultures), and flour composition (especially protein, carbohydrate, and ash content)^11^.

Lactobacilli have a long history of being used as biological preservatives. It has been proven that using sourdough containing these bacteria is the best method for preserving bread from mold spoilage due to the action of lactic acid bacteria in producing organic acids (lactic and acetic acid) during fermentation. Furthermore, the acidification of the dough has significant effects on the quality characteristics of bread, such as texture and volume. Among the various species of lactobacilli used in sourdough, *Lactobacillus plantarum* and *Lactobacillus brevis* have been shown to have beneficial effects on bread properties^12^. Cetin-Babaoglu et al. (2023) isolate sourdough starters (produced with *Lactobacillus fermentum* and *Lactococcus lactis*) and use them in the production of dephytinized wheat bran enriched bread. They reported that the use of *L. fermentum* in the dough preparation led to an improvement in the quality characteristics of the produced bread^13^. Therefore, considering the global community’s need to reduce waste and enhance the quality and shelf life of bakery products, the aim of this study was to investigate the impact of using sourdoughs from various bacteria and their usage levels in the formulation on the physicochemical, microbiological, and sensory characteristics of gluten-free bread samples prepared from rice flour.

## Materials and methods

### Materials

In this study, to prepare gluten-free bread, completely debranized Fajr rice flour (without pigment) from rice production plants in Golestan province, Iran (with α-amylase 165 U/mL), salt, and sugar from the local market (Tabriz, Iran), Ringer’s tablets (Merck, Germany), baker’s yeast (*Saccharomyces cerevisiae*) (Pakmaya, Turkey), lyophilized ampoules of *L. fermentum* and *L. plantarum* (Iranian Research Organization for Science and Technology), and de Man, Rogosa and Sharpe (MRS) agar and broth and Sabouraud dextrose agar (SDA) culture media (Merck, Germany) were used.

### Preparation of sourdough using specific starter

To prepare the sourdough (in three repetitions), initially, dough was prepared at a dough yield (DY) of 200 or 300% by mixing flour and water. Then strains of *L. fermentum* and *L. plantarum*, which have been proven to have GRAS (Generally Recognized as Safe) properties, were cultured in MRS Broth at a 1% weight ratio at 37 °C for 24 h to activate. The lactobacilli were grown until they reached 10^7^ cfu/g (based on the McFarland method). To isolate fresh microbial cells, the produced biomass was separated from the culture medium using a centrifuge (Vs-180cf, Germany) at 5000 g for 15 min at 4 °C^14,15^. In this study, dough yields of 200% and 300% (Eq. 1) were selected for preparing the sourdough. After preparing the sourdough with the desired dough yield, the bacterial strains were inoculated into the dough separately (at a concentration of 10⁷ (CFU/g) of dough) and incubated for 24 h at 30 °C.

1 $$\:\text{D}\text{o}\text{u}\text{g}\text{h}\:\text{y}\text{i}\text{e}\text{l}\text{d}=\frac{F+W}{F}\times\:100$$

Where F and W are respectively equal to the amount of flour and water used in the dough formulation.

###  Preparing rice-based gluten-free bread

#### Preparing rice-based gluten-free bread

For the preparation of gluten-free bread based on rice flour, the proposed formulation in the method by Gharekhani et al. (2021) was used with some modifications^14^. In this study, control bread was made without using sourdough, and for other treatments, sourdough was substituted in the dough formulation at levels of 10, 20, and 30% in place of flour. Initially, 80% of the water used was mixed with all the required salt (2% by weight of flour (WF)) and then mixed with all the rice flour for 3 min in a mixer. Subsequently, all the ingredients including sugar (5% WF), dried milk (5% WF), guar gum (3% WF), along with sourdough, oil (5% WF), egg (12% WF), activated yeast (3% WF), and the remaining water were added to the flour samples. Then, 225 g of the dough was poured into baking molds made of galvanized sheets (with dimensions of 15 × 8.5 × 5.7 cm). To prevent the dough from sticking to the container, the containers were first greased with a type of edible oil (Sunflower oil). The final proofing took place for 75 min in a proofing chamber at a temperature of 30 °C and a relative humidity of 75%, and baking was done at a temperature of 225 °C for 30 min. Finally, the breads were cooled at room temperature for 1 h and then packaged in polyethylene bags. The treatments were carried out with three replications and their abbreviations are given in Table 1.

**Table 1:** Treatments and abbreviations used in this study.

Number	Abbreviation	The type of bacteria used in sourdough	Dough yield (%)	The amount of sourdough (%)
1	Control	–	–	–
2	LF.200.SD10	*Lactobacillus fermentum*	200	10
3	LF.200.SD20	*Lactobacillus fermentum*	200	20
4	LF.200.SD30	*Lactobacillus fermentum*	200	30
5	LF.300.SD10	*Lactobacillus fermentum*	300	10
6	LF.300.SD20	*Lactobacillus fermentum*	300	20
7	LF.300.SD30	*Lactobacillus fermentum*	300	30
8	LP.200.SD10	*Lactobacillus plantarum*	200	10
9	LP.200.SD20	*Lactobacillus plantarum*	200	20
10	LP.200.SD30	*Lactobacillus plantarum*	200	30
11	LP.300.SD10	*Lactobacillus plantarum*	300	10
12	LP.300.SD20	*Lactobacillus plantarum*	300	20
13	LP.300.SD30	*Lactobacillus plantarum*	300	30

L.F: *Lactobacillus fermentum*, L.P: *Lactobacillus plantarum*, SD: sourdough.

### Sourdough analysis

#### pH

10 g of dough were homogenized with 90 ml of distilled water, and then the pH of the dough was measured using an electronic pH meter (Milwaukee Martini, Italy)^16^.

#### Determining the amount of lactic acid.

The lactic acid content was determined using HPLC (Knauer, Germany) following a method described by Hadaegh et al. (2017). Briefly, 10 g of the samples were homogenized with 90 ml of distilled water. Then, 10 ml of this mixture was added to 5 ml of 0.1 mM HClO4 solution and centrifuged in a centrifuge (Vision, Germany) at 4000 g for 15 min. The supernatant was acidified to pH = 3 with 0.1 mM HClO4 solution and brought to a final volume of 25 ml with distilled water. After keeping for 30 min in ice water, the solutions were filtered through a cellulose acetate filter with a pore size of 0.22 μm (Millipore, Spain) and injected into the HPLC system. The column used for this study was 5 μm, 150 × 4.6 ml, with an isocratic mobile phase of 0.5 mM HClO4, a flow rate of 0.5 ml/min, and a column temperature of 35 °C with an automatic injection system, and finally, an injection volume of 100 µL^12^.

#### Determining the number of lactic acid bacteria

10 g of sourdough were homogenized in 90 ml of 0.15 M NaCl, and after preparing various dilutions in phosphate-buffered saline (PBS), they were cultured on MRS agar. The cultures were incubated for 48 h at a temperature of 30 °C, and the bacterial count was conducted^14^.

### Analysis of rice-base gluten-free bread

####  pH

The pH level of the bread, similar to the dough’s pH, was determined by immersing the electrode of an electronic pH meter into a suspension consisting of 10 g of homogenized bread (a homogeneous mixture of the crust and crumb) and 90 ml of water^16^.

#### Moisture content

The moisture content of the bread was measured according to the standard method AACC 44 − 15. The moisture of the fresh bread crumb, two hours after baking, was determined using an electric oven (Memmert, Germany) at a temperature of 100–105 °C^17^.

#### Baking loss

The weight of the dough pieces and the weight of the bread samples after baking and cooling for 2–3 h were measured, and the percentage of baking loss was calculated using Eq. 2^14^.

2 $${\text{Baking loss }}\left( \% \right){\text{ }} = \:\frac{{({\text{Weight}}\:{\text{of}}\:{\text{dough}}\:{\text{piece}}\: - \:{\text{Weight}}\:{\text{of}}\:{\text{bread}}\:{\text{after}}\:{\text{baking}})\:}}{{\left( {Weight\:of\:dough\:piece} \right)}} \times \:100$$

#### Firmness

The texture of the produced breads was evaluated using the Texture Profile Analysis (TPA) method, 2 h after baking. For this purpose, cubic pieces measuring 2 × 2 × 2 cm were first prepared from the crumb of the bread samples. Their texture was then measured and evaluated using a Brookfield Texture Analyzer (Model LFRA-4500, USA). The maximum force required for a cylindrical probe with a diameter of 25 mm to penetrate up to 50% of the initial height at a speed of 10 mm/min was considered as the firmness of the bread and evaluated^18^.

#### Bread crust color indices

For the color analysis of the samples, a computer image processing method was used. In this method, photographs of the bread surface were taken using a digital camera (for photography, the samples were placed inside a chamber with a black background, and to illuminate the space, energy-efficient fluorescent lamps were used; the angle between the camera lens and the light source axis was about 45° to ensure the reflected light to the camera was not from the light source but from the samples; also, the distance of the samples from the camera was considered to be 30 cm) and after transferring to a computer, the color indices (L*, a*, and b*) were determined using Photoshop version (CC 2018, 19.1). By calibrating the value of these parameters using color cards, the actual level of these parameters was determined. The L* index represents the lightness level of the sample and ranges from zero (pure black) to 100 (pure white). The a* index indicates how close the color of the sample is to green or red colors, with a range from − 120 (pure green) to + 120 (pure red). Finally, the b* index shows how close the color of the sample is to blue or yellow colors, with a range from − 120 (pure blue) to + 120 (pure yellow)^14^.

#### The number of mold and yeast

After the breads were made, a test to determine the number of molds and yeasts on the breads was conducted on the first and fourth days after production. For this purpose, some of the bread samples on the mentioned days were placed inside sterile Stomacher bags near a flame, then the sterile bags were placed in a Stomacher and homogenized for 4 min. At this stage, the sample was thoroughly mixed and turned into a uniform fragments. From the resulting sample fragments, 10 g were weighed and mixed in a sterile beaker with 90 ml of sterile Ringer’s solution. Using a pipette, 0.1 ml of the serial dilutions prepared was transferred to two separate plates, each containing Sabouraud Dextrose Agar (SDA) culture medium. The inoculated plates were incubated at 25 °C for 5 days and checked between two to five days after incubation. Plates with fewer than 150 colonies were counted, and the dilution factor and the number 10 were multiplied^19^.

#### Sensory analysis

The sensory evaluation was conducted by 10 semi-trained panelists familiar with sensory evaluation techniques. The baked samples were provided to the panelists 2 h after baking. Sensory characteristics of the bread, including shape and form, top surface characteristics, bottom surface characteristics, porosity, firmness and softness of texture, chewability, and aroma, taste and flavor, which respectively had weighting factors of 4, 2, 1, 2, 2, 3, and 3, were assessed. The evaluation scale for these characteristics ranged from very bad (1), bad (2), average (3), good (4), and very good (5). With this information, overall acceptance was calculated using Eq. 3^20^.

3 $$\:Q=\frac{\sum\:(P\times\:G)\:}{\sum\:P}$$

Where Q = overall acceptance (number of quality of production samples), P = attribute rating coefficient and G = attribute evaluation coefficient.

### Statistical analysis

In this research, all tests were conducted in triplicate. The data obtained from the tests were analyzed statistically using a completely randomized design with the Statistical Analysis System (SAS) software, Version 9.4 (SAS Institute Inc., Cary, NC, USA, https://support.sas.com/downloads/browse.htm?cat=63). Mean comparisons were made using Duncan’s multiple range test at a 95% confidence level, and Excel (2007) was utilized for creating the charts.

## Results and discussion

### The effect of bacteria type and dough yield on characteristics of sourdough

Table 2 shows that the maximum pH of sourdough is correlated with samples that used a *L. fermentum* starter with 300% and 200% yield for preparation. The data generally showed that the pH levels were higher in samples with *L. fermentum* than in those with *L. plantarum*, this is likely because there is less decomposition of polysaccharide compounds in the formulation, leading to reduced production of acidic compounds. Conversely, the sample made with a 200% yield *L. plantarum* starter registered the highest concentration of lactic acid among all samples (*p* < 0.05), and an increase in dough yield was associated with a decrease in lactic acid concentration (Table 2). The findings also showed that the sample containing *L. plantarum* and *L. fermentum* starters with a dough yield of 300% had the lowest bacterial count in the dough. The analysis also confirmed that a rise in dough yield led to a reduction in the dough’s bacterial population (*p* < 0.05). Dough yield defined as 100 parts of flour plus the amount of water (in parts) used for hydration. Therefore, the higher the amount of water (dough yield), the lower the amount of lactic acid bacteria in the sourdough^21^.

The pH levels of both dough and bread play a critical role in the breakdown of phytic acid. The solubility of phytates, which form complexes with cations, is influenced by the pH level, the types of cations present, and their concentrations^22^. Excessively acidic conditions can adversely affect the quality of bread, potentially leading to a lack of flavor, a decrease in bread volume, and an increase in the bread’s firmness^23^. Torrieri et al. (2014) documented the pH of sourdoughs created using lactic acid bacteria as approximately 4.00, noting that differences between these values and those of the current study could be attributed to alterations in the sourdough’s formulation^24^. Factors such as fermentation duration and temperature, flour type, dough performance, and the bacterial strains utilized can impact the sourdough’s biochemical properties, including its pH and acidity levels^11^.

**Table 2:** The effect of bacteria type and dough yield on sourdough characteristics.

Characteristics	Type of sour dough			
	L.F.200	L.F.300	L.*P*.200	L.*P*.300
pH	4.53 ± 0.30^ab^	4.60 ± 0.32^a^	3.79 ± 0.10^c^	^bc^3.88 ± 0.25
Lactic acid (mg/100 g dry basis)	520.33 ± 9.26^b^	129.00 ± 5.41^d^	620.33 ± 9.79^a^	^c^169.00 ± 6.16
count bacteria (Log cfu /g)	9.84 ± 0.20^a^	8.80 ± 0.10^b^	9.46 ± 0.30^a^	8.33 ± 0.20^b^

The data are the mean of three replicates ± standard deviation and similar lowercase letters in each row indicate the lack of significance at the 5% level. L.F.200 (sour dough containing *L. fermentum* with 200% dough yield), L.F.300 (sour dough containing *L. fermentum* with 300% dough yield), L.P.200 (sour dough containing *L. plantarum* with 200% dough yield) and L.P.300 (sour dough containing *L. plantarum* with 300% dough yield).

### Bread pH

**Fig. 1:**
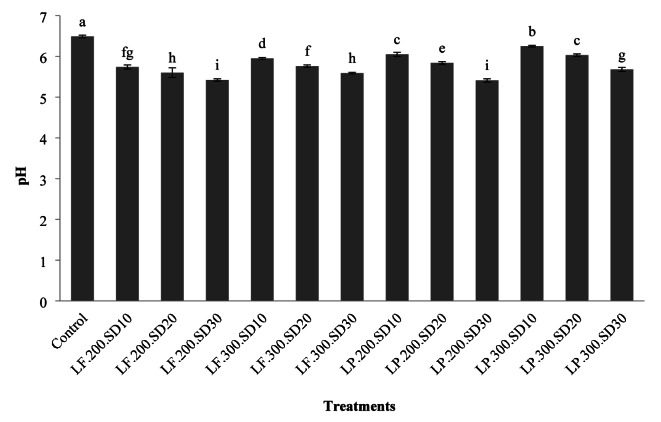
The effect of the type of formulation on the pH of rice-base gluten-free bread (the numbers in each column are the mean of three replicates ± standard deviation and the same lowercase letters on each column indicate the lack of significance at the 5% level).

The results depicted in Fig. 1 indicated that the highest pH levels were associated with the control sample, while the lowest pH levels were linked to the sample derived from sourdough containing *L. plantarum* and *L. fermentum*, with a dough yield of 200% and 30% sourdough. Additionally, the research showed that incorporating more sourdough into the bread recipes decreased the pH values of the samples. Conversely, a higher dough yield was associated with higher pH values, with *L. fermentum* proving to be more effective at lowering the pH in the breads produced.

The decrease in pH with an increased percentage of sourdough can be attributed to the rise in acids produced in the dough and the subsequent reduction in the pH of the produced breads. Aplevicz et al. (2013) demonstrated that the use of sourdough led to a reduction in the pH of the produced breads^25^. Banu et al. (2011) and Tariverdi and Gharekhani (2025) also demonstrated that using sourdough resulted in a decrease in the pH levels of breads made from rye and corn flour, respectively^26,27^. Oshiro et al. (2020) also determined that an increase in the percentage of sourdough and process time decreased the pH levels of breads, aligning with the results of this study^28^. Katsi et al. (2021) also found that using sourdough led to a reduction in the pH levels of the produced breads^29^. Sourdough bacteria can consume the carbohydrates in flour for their metabolism, resulting in the production of acetic and lactic acids, which ultimately lowers the pH and increases acidity^30^.

According to the Iranian national standard, the pH range of bread is from 4.6 to 6.0^31^. In the present study, four samples were tested: the control, a sample prepared with 10% and 20% sourdough containing *L. plantarum* with a dough yield of 300%, and a sample prepared with 10% sourdough containing *L. fermentum* with a dough yield of 300%. All four samples were found to be above this range.

### Bread moisture

**Fig. 2:**
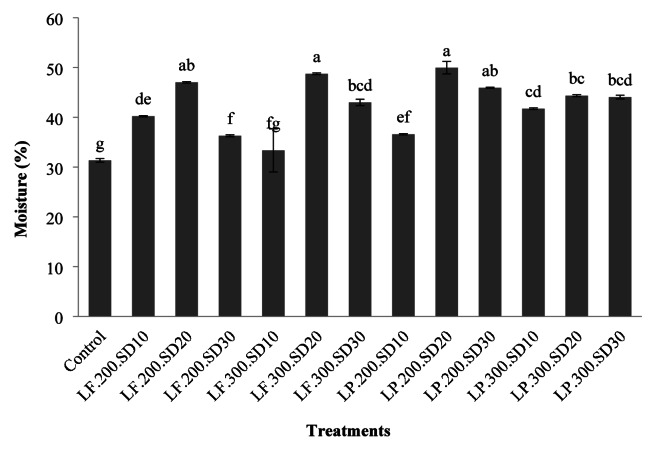
The effect of the type of formulation on the moisture content of rice-base gluten-free bread (the numbers in each column are the mean of three replicates ± standard deviation and the same lowercase letters on each column indicate the lack of significance at the 5% level).

Duncan’s method of mean comparison (Fig. 2) revealed that incorporating sourdough, except the 10% *L. fermentum* sourdough sample at a 300% yield, resulted in higher moisture levels in the bread produced. It was noted that an increment in sourdough percentage initially led to an increase in moisture, which then diminished, and moisture levels did not exhibit a consistent pattern with dough yield changes. In certain instances, a rise in dough yields enhanced moisture content, whereas, in others, it led to a reduction. The enhancement in moisture content within sourdough-based breads can be linked to the enzymatic actions of the sourdough bacteria and the generation of exopolysaccharides that aid in moisture retention^32^. Di et al. (2018) found that sourdough utilization in steamed bread preparation preserved more moisture than the control samples^33^. Several studies have highlighted the positive impact of sourdough on bread’s moisture retention and the postponement of bread staling, attributed to the fermentative by-products and proteolytic actions of these bacteria^23,24^. Similarly, Gharekhani et al. (2021) presented findings that aligned with this study, showing an increase in moisture content with sourdough use compared to control samples^14^.

The decrease in moisture content in some samples, with a greater increase in the percentage of sourdough in the formulation, can also be attributed to the higher activity of enzymes secreted by sourdough bacteria and the decomposition of compounds with a greater water retention capacity. According to Ronie et al. (2023), the acceptable range for the typical moisture content of bread for day-one storage is between 35% and 45%^34^. On the other hand, according to the findings of Jitrakbumrung and Therdthai (2014), the use of sourdough can lead to an increase in bread moisture content, sometimes up to 5.6%. Consequently, the moisture content of all our breads fell within this range^35^.

### Bread baking loss

**Fig. 3:**
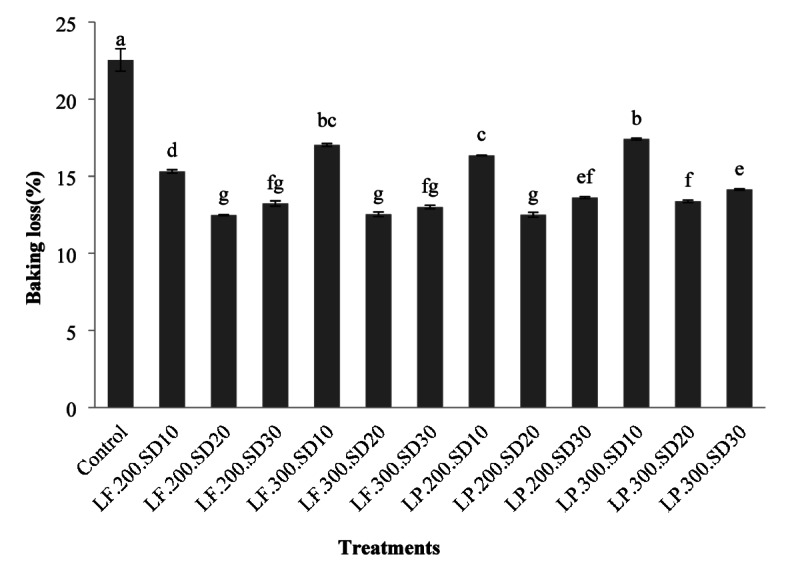
The effect of the type of formulation on the baking loss of rice-base gluten-free bread (the numbers in each column are the mean of three replicates ± standard deviation and the same lowercase letters on each column indicate the lack of significance at the 5% level).

The findings showed that all samples containing sourdough had lower baking loss than the control sample. As the percentage of sourdough increased, the baking loss initially decreased and then increased. Additionally, with an increasing dough yield, the baking loss increased in samples prepared with sourdough containing *L. plantarum*. However, in samples prepared with sourdough containing *L. fermentatum*, this only occurred in the sample containing 10% sourdough (Fig. 3).

According to Wolter et al. (2014), the addition of sourdough fermented with *L. plantarum* FST 1.7 significantly reduced baking loss in all gluten-free breads, except teff bread, compared to control bread. The reduction in baking loss can be attributed to the reaction of compounds produced by lactic acid bacteria (such as various oligosaccharides) and their binding with water, which prevents water from escaping during baking^36^. Similarly, Gharekhani et al. (2021) observed a reduction in baking loss when sourdough was used^14^. Nami et al. (2019) found that increasing sourdough percentages led to lower baking losses in millet-based breads^37^. Schoenlechner et al. (2023) reported that adding more water to the dough of sorghum and quinoa bread resulted in lower baking losses due to increased moisture release during baking^38^. Cappa et al. (2016) also found that sourdough’s use in bread preparation resulted in greater moisture retention and thus, lower baking losses due to enhanced moisture preservation throughout the baking process^39^.

### Bread firmness

**Fig. 4:**
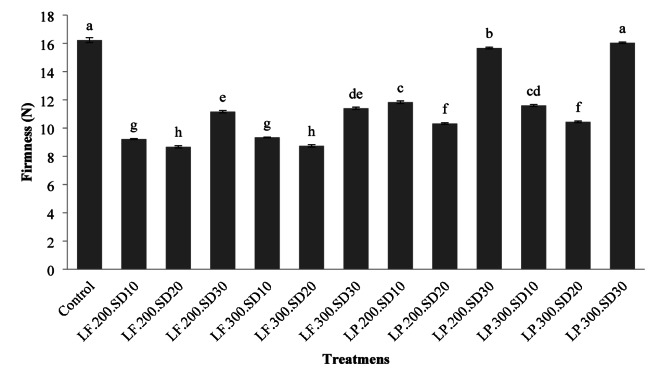
The effect of the type of formulation on the firmness of rice-base gluten-free bread (the numbers in each column are the mean of three replicates ± standard deviation and the same lowercase letters on each column indicate the lack of significance at the 5% level).

Figure 4 indicated that adding sourdough to the bread mix led to a reduction in the bread’s firmness, with firmness decreasing until sourdough made up 20% of the formulation and then started to increase. Additionally, except when utilizing *L. plantarum* at 30% sourdough, the impact of dough yield on firmness was not statistically significant (*p* > 0.05). The decrease in firmness can be linked to starch molecules breaking down due to enzymes from lactic acid bacteria, altering starch’s retrogradation characteristics and thus slowing the staling rate. The increased firmness of bread is likely due to compounds formed from further starch decomposition and the predominance of A-type granules in the dough. These crystals have the ability to retain less moisture during baking^40^. According to Sanz-Penella et al. (2012), bread firmness increases with the addition of yeast to the wheat flour mixture, which is consistent with the observations in this Section^41^. Similarly, Katsi et al. (2021) showed that too much sourdough leads to a firmer bread texture^29^. On the other hand, Torrieri et al. (2014) observed that sourdough usage tends to soften bread^24^. Xu et al. (2019) explained that the softer bread texture with more sourdough addition is due to the increased release of CO_2_, thymol, and hydrogen peroxide, which expands the bread volume and reduces its firmness^42^. Furthermore, the decrease in bread firmness can also be attributed to the activation of flour proteolytic enzymes, which enhance protein solubility and increase alpha-amylase activity. This, in turn, leads to greater moisture absorption of polysaccharides^11^. Casado et al. (2018) also noted an increase in the chewiness and firmness of bread made from sourdough^43^. Similarly, Siepmann et al. (2019) reported that bread firmness increases with sourdough addition and higher fermentation temperatures^44^.

### Bread color indices

One of the qualitative characteristics of food products is their color, which plays a significant role in the acceptability of food products nowadays. If a food product lacks the appropriate color, one of its visual characteristics, it will face a significant decrease in its market value. Other quality characteristics such as aroma, taste, texture, etc., are parameters that are judged after consuming the food product, possibly after buying and consuming it once^45^.

Color indices related to the bread crust are presented in Table 3. As evident, with an increase in the percentage of sourdough in the bread formulations, the L* index of the produced samples increased, but the a* and b* indices decreased. Ultimately, *L. plantarum* was more successful in increasing the L* index and decreasing the a* and b* indices. An increase in dough yield also led to a reduction in the trend of changes in color indices except for L.P. a* values (Table 3). According to the results of this section, Torrieri et al. (2014) also stated that the use of sourdough in bread making leads to an increase in the L* index and a decrease in the a* and b* indices^24^. These researchers associated browning of the crust with caramelization and Maillard reactions, which belong to the category of non-enzymatic or non-oxidative browning. The crust color also depends on the physical and chemical properties of the raw dough (i.e., water content, pH, reducing sugars, and amino acid content) as well as the operational conditions applied during baking (such as temperature, air velocity, relative humidity, heat transfer conditions^46,47^. The increase in brightness of the breads with the addition of sourdough can be attributed to the consumption of some fermentable sugars and amino acids by the bacteria present in the sourdough before the Maillard reaction occurs^48^. Codină et al. (2021) also mentioned that the use of sourdough up to a certain amount led to an increase in the L* index of the samples^49^. Ronie et al. (2023) also attributed the increased brightness in the crust of gluten-free breads made with Bario rice flour to the lower sugar content in the rice bread formulation, which plays a role in the Maillard reaction^34^.

**Table 3:** The effect of sample type on bread crust color indices.

Type of sample	Color index		
	L^*^	a^*^	b^*^
Control	63.67 ± 0.17^g^	13.33 ± 0.10^a^	59.00 ± 0.21^a^
LF.200.SD10	68.33 ± 0.22^e^	9.65 ± 0.31^c^	57.33 ± 0.33^b^
LF.200.SD20	70.33 ± 0.67^d^	7.47 ± 0.19^e^	41.00 ± 0.67^g^
LF.200.SD30	74.00 ± 0.33^b^	3.33 ± 0.22^h^	36.00 ± 0.41^i^
LF.300.SD10	65.00 ± 0.47^f^	11.71 ± 0.17^b^	60.33 ± 0.59^a^
LF.300.SD20	68.67 ± 0.47^e^	9.70 ± 0.09^c^	52.33 ± 0.17^cd^
LF.300.SD30	70.33 ± 0.15^d^	5.43 ± 0.13^g^	46.62 ± 0.62^e^
LP.200.SD10	70.65 ± 0.17^d^	8.00 ± 0.27^de^	51.00 ± 1.06^d^
LP.200.SD20	72.50 ± 0.88^c^	5.67 ± 0.09^g^	36.00 ± 0.34^i^
LP.200.SD30	75.80 ± 0.18^a^	-1.00 ± 0.50^i^	32.50 ± 0.26^j^
LP.300.SD10	65.43 ± 0.33^f^	10.33 ± 0.13^c^	52.67 ± 0.42^c^
LP.300.SD20	69.41 ± 0.66^de^	8.76 ± 0.21^d^	43.72 ± 0.23^f^
LP.300.SD30	72.40 ± 0.67^c^	6.71 ± 0.11^f^	37.52 ± 0.71^h^

The data are the mean of three replicates ± standard deviation and similar lowercase letters in each row indicate the lack of significance at the 5% level.

#### Mold and yeast counts

The mold and yeast counts related to the produced breads are presented in Table 4. As shown, the use of sourdough has inhibited further activity of mold and yeast, and an increase in storage time, dough yield, and a decrease in the percentage of sourdough led to an increase in mold and yeast counts (*p* < 0.05). The highest count of mold and yeast was observed in the control breads after four days of storage, and the lowest count of mold and yeast was seen in the breads made from 30% sourdough containing *L. fermentatum* or *L. plantarum* with a 200% dough yield on the first day after production.

Bread is generally considered a sterile product due to the high temperatures of processing (above 170 °C) and is thought to be free of microbial contamination; primarily, the presence of yeast and mold in this product is due to contamination after baking. The reduction in mold and yeast counts in bread made from sourdough can be attributed to the higher acidity and lower pH associated with them^29^. Mohsen et al. (2016) examined the quality characteristics of Egyptian Balady bread using sourdough containing (2% *Saccharomyces cerevisiae* plus 1, 2, or 3% *L. plantarum*). The results indicated an increase in organic acids, antimicrobial activity, and a decrease in pH during the preparation of different sourdough samples^50^. Movahhed (2021) also showed that an increase in the percentage of sourdough in Sangak bread formulation led to a reduction in mold and yeast counts due to increased acidity, aligning with the findings of this Section^30^.

**Table 4:** The effect of sample type on bread mold and yeast counts (log CFU/g).

Type of sample	Storage time (day)	
	1	4
Control	4.41 ± 0.03^aB^	7.95 ± 0.01^aA^
LF.200.SD10	1.76 ± 0.31 ^dB^	4.83 ± 0.12^fA^
LF.200.SD20	1.74 ± 0.29 ^dB^	4.19 ± 0.10^hA^
LF.200.SD30	0.72 ± 0.25 ^eB^	3.75 ± 0.03^iA^
LF.300.SD10	3.69 ± 0.02^bB^	7.09 ± 0.01^bB^
LF.300.SD20	3.68 ± 0.02^bB^	6.78 ± 0.01^cA^
LF.300.SD30	3.39 ± 0.14^cB^	5.59 ± 0.03^dA^
LP.200.SD10	1.75 ± 0.22 ^dB^	4.54 ± 0.02^gA^
LP.200.SD20	1.70 ± 0.22 ^dB^	4.50 ± 0.03^gA^
LP.200.SD30	0.72 ± 0.25^eB^	3.68 ± 0.12^iA^
LP.300.SD10	3.44 ± 0.03^cB^	6.79 ± 0.02^cA^
LP.300.SD20	3.32 ± 0.17^cB^	6.76 ± 0.02^cA^
LP.300.SD30	3.22 ± 0.15^cB^	5.01 ± 0.03^eA^

The data are the mean of three repetitions ± standard deviation and the same lowercase letters for each column and the same uppercase letters for each row indicate lack of significance at the 5% level.

#### Overall acceptance

**Fig. 5:**
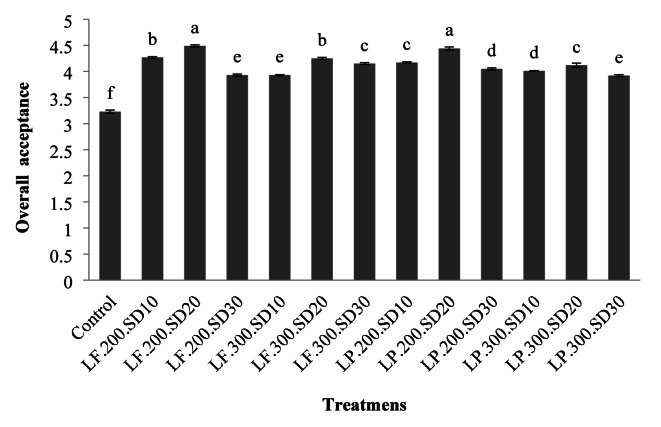
The effect of the type of formulation on the overall acceptance of rice-base gluten-free bread (the numbers in each column are the mean of three replicates ± standard deviation and the same lowercase letters on each column indicate the lack of significance at the 5% level).

Figure 5 illustrated that with an increase in sourdough percentage up to 20% in the bread formulations, the scores for overall acceptance from sensory panelists increased and then decreased. In most samples, except for those containing 30% sourdough from *L. fermentatum*, an increase in dough yield led to a decrease in overall acceptance. Campo et al. (2016) attributed the increased overall acceptance of samples containing sourdough to their improved aroma, flavor, and more appealing appearance^51^.

The decrease in overall acceptance for samples made with a higher concentration of sourdough could be due to the sharp odor resulting from the activity of lactic acid bacteria. Codină et al. (2021) also showed that an increase in sourdough initially raised the overall acceptance scores from evaluators and then decreased them, aligning with the findings of this section. These researchers attributed the increase in overall acceptance with more sourdough to the retention of sugars and amino acids during lactic acid bacteria activity, which participate in the Maillard reaction, creating a desirable taste, aroma, and color^49^. Zhao and Ganzle (2016) stated that lactic acid bacteria, due to their low proteolytic activity, contribute to the accumulation of amino acids during fermentation, creating suitable flavors and aromas that improve the overall acceptance of the samples^52^.

## Conclusions

The results of this study aimed to enhance the physicochemical, microbial, and sensory properties of gluten-free bread made from rice flour using sourdoughs containing Lactobacillus fermentum and plantarum starters. The study found that the pH levels in the sourdoughs of samples containing *L. fermentum* were higher than those with *L. plantarum*. Additionally, increasing the dough yield resulted in a decrease in lactic acid and the number of bacteria in the sourdoughs. As the percentage of sourdough in gluten-free bread based on rice flour increased, the pH decreased, but the use of sourdough led to higher moisture content compared to the control sample. Overall, the use of sourdough containing these starters improved the physicochemical, microbial, and sensory properties of gluten-free bread based on rice flour. It can be concluded that using 20% sourdough obtained from the activity of these two bacteria and a yield of 200% resulted in the production of bread with the best quality according to evaluators. While these results are promising, further research is needed to explore the potential health benefits of gluten-free bread made with rice flour and sourdough. This includes investigating antioxidant properties and other beneficial compounds.

## Data Availability

The data supporting the findings of the study are available and will be provided upon reasonable request by the corresponding author.
